# Reimagining violence: Social representations, emotions and the politics of resistance

**DOI:** 10.1111/bjso.70148

**Published:** 2026-11-05

**Authors:** Subas Amjad Ali, Anderson da Silveira, Kata Bődi, Hanan Omar Abdallah Zaid Alkilani, Sara Bigazzi

**Affiliations:** ^1^ Department of Social and Organizational Psychology University of Pécs Pécs Hungary; ^2^ Department of Communication and Social Research Sapienza University of Rome Rome Italy; ^3^ Laboratory of Social Psychology of Communication and Cognition (LACCOS) Federal University of Santa Catarina Florianópolis Brazil

**Keywords:** affective polyphasia, emotions, resistance, social representations, violence

## Abstract

Political violence occupies a contested space shaped by power asymmetries, colonial legacies and competing moral frameworks. Drawing on Social Representations Theory (SRT) and affective polyphasia, this study examines how individuals evaluate the legitimacy of violence as resistance and how emotional repertoires structure these judgements, using a mixed‐methods instrument administered to 444 participants across 64 countries. Network analysis identified anger and hope as central, co‐occurring emotions with complementary temporal orientations–anchoring resistance in past injustice and future possibility. Factor analysis revealed three emotional repertoires (transformative, self‐evaluative, threat) and three dimensions of violence. Emotions were associated with activism indirectly through distinct pathways, varying by type: transformative emotions required cognitive anchoring, self‐evaluative emotions involved identity processes and threat emotions reflected ambivalence, with material damage emerging as a key mediator linking systemic critique to action. Cluster analysis identified five emotional profiles varying by geopolitical region and ideology. Findings extend SRT, refine collective action models and challenge linear emotion–action assumptions.

## INTRODUCTION

Revolutionary violence occupies a contested position within political discourse, oscillating between narratives of disruption and liberation, a tension itself produced by competing social representations rather than a fixed moral reality. Violence is increasingly conceptualized not as an isolated act but as a relational phenomenon embedded within asymmetrical power structures and normative orders (Barkawi & Laffey, 2006; Becker & Tausch, 2017; Bilali & Vollhardt, 2019), with collective action research distinguishing normative protest, which operates within accepted political rules, from non‐normative forms that transgress them (Becker & Tausch, 2017). What counts as legitimate action is not fixed but defined relative to historically constituted systems of authority, so evaluations of violence are shaped by broader historical and institutional frameworks, not the act alone.

These normative frameworks are embedded within global hierarchies shaped by colonial formations and Eurocentric narratives of political modernity (Barkawi & Laffey, 2006; Quijano, 2000), persisting through symbolic and epistemic structures that define legitimacy and resistance (Smith, 2019). Foundational moral narratives, such as the Second World War in Western societies, normalize state violence while pathologizing resistance from subordinate positions, functioning as living memory that shapes political legitimacy and present‐day responses to injustice (Liu & Hilton, 2005); collective victimhood can likewise function as a psychological resource sustaining resistance across generations (Selvanathan et al., 2023). Fanon (1967) highlighted violence's transformative character in decolonial struggles, a tradition extended by theorizing critical consciousness as a politically generative process of responding to oppression from decolonial and feminist standpoints (Cavazzoni et al., 2023), situating emotions as politically constituted responses to structural violence within a critical psychology tradition oriented towards social justice (Fox et al., 2009).

Within social psychology, early work emphasized individual aggression (Berkowitz, 1989), later shifting towards intergroup dynamics, dehumanization and structural violence in protracted conflicts (Christie et al., 2001; Mackie et al., 2000; Staub, 1990; Vollhardt, 2012). Recently, collective action research has identified identity, emotion and efficacy as central motivational drivers (Van Zomeren et al., 2008). More recently, scholars have proposed a systems perspective on emotion in collective action, emphasizing the dynamic, interdependent, and recursive relationships between emotions, appraisals and political engagement, moving beyond linear models towards understanding emotions as embedded within broader socio‐political systems (Bou Zeineddine & Leach, 2021; Leach et al., 2024; Leach & Bou Zeineddine, 2021). When institutional channels are perceived as closed or illegitimate, protest becomes a dialogical process between citizens and power holders: Becker and Tausch (2017) show that support for radical tactics stems from low efficacy and contempt for authorities rather than anger alone, and disruptive tactics may emerge to create leverage and re‐open dialogue when claims are repeatedly excluded.

Yet these evaluations are embedded within asymmetrical power structures that functionally concentrate and legitimize violence for dominant actors (Barkawi & Laffey, 2006; Bilali & Vollhardt, 2019): state coercion is naturalized through discourses of ‘security’, while resistance by marginalized groups is frequently pathologized as irrational or deviant (Bou Zeineddine & Vollhardt, 2024; Vollhardt, 2020), obscuring the historical role of structural violence in constituting the order later framed as ‘disrupted’ (Galtung, 1969; McClintock, 2013). Rather than judging the moral status of violence, the present study examines how its legitimacy is evaluated, drawing on Hodges and Geyer's (2006) concept of value pragmatics, whereby social actors navigate ‘heterarchies’ of competing values such as order, justice and solidarity. Evaluations of legitimacy are thus perspectival achievements, emerging through the mobilization and prioritization of competing moral vocabularies and historical memories.

This study uses the paradigm of Social Representations Theory (Moscovici, 2008), which conceptualizes violence not only as a behaviour but as a representational object, socially constructed, historically anchored, morally negotiated and embedded within symbolic systems (Marková, 2003; Wagner, 1998). Through anchoring and objectification, unfamiliar political acts are interpreted by linking them to existing moral frameworks and transforming abstract conflicts into concrete images, actors and events (Bauer & Gaskell, 1999; Moscovici, 2008). In contentious politics, these representations are often anchored in historically recognizable repertoires of protest, strikes, uprisings, sabotage, symbolic iconoclasm, providing culturally available templates through which resistance becomes intelligible (Hakoköngäs & Sakki, 2016; Tilly, 1978).

Recent empirical applications demonstrate how these processes operate in conflict contexts. Olumba (2023) showed that parties in Nigeria's Middle Belt anchor present eco‐violence to historical land contestations, naturalizing collective memories that sustain violent redress cycles, while Barreiro et al. (2017) found that competing representations of Argentina's ‘Conquest of the Desert’ legitimize state violence while alternative representations contest these narratives as resistance. These studies underscore that political violence is not a stable category but a representational object whose meaning is continuously negotiated through historically situated moral vocabularies.

Social representations theory highlights that multiple and even contradictory systems of knowledge can coexist within the same representational field in a state of cognitive polyphasia, allowing individuals to draw upon different rationalities depending on context or political positioning (Howarth et al., 2014; Jovchelovitch, 2008; Marková, 2015; Moscovici, 2008). Bruno and Barreiro (2021) found that adolescents simultaneously hold conventional and unconventional political representations, with polyphasia manifesting as selective prevalence depending on discursive context; asymmetries of power can render some representations dominant while relegating others into ‘nothingness’, with implications for identity politics and collective memory (Barreiro et al., 2017).

Social representations are not merely cognitive descriptions but forms of social action (Batel, 2012): cognitive polyphasia reflects the coexistence of heterogeneous repertoires of meaning‐making through which actors interpret and justify social phenomena (Bruno & Barreiro, 2021), emerging through dynamic interactions between different knowledge systems and emotional tensions (de‐Graft Aikins, 2012). This emotional co‐existence resonates with a systems perspective on collective emotion, in which emotions are not isolated responses but form interdependent networks that mutually activate, suppress or transform one another in the context of political action (Bou Zeineddine & Leach, 2021; Leach & Bou Zeineddine, 2021). Emotions thus function as a modality of knowledge, mediating how actors interpret threats, possibilities and moral boundaries, and representational hybridity becomes a political mechanism through which actors negotiate legitimacy.

In contexts of political conflict, polyphasia can manifest simultaneously at several levels: representational (coexistence of different moral frameworks for interpreting violence), affective (coexistence of distinct emotional repertoires mobilized in relation to protest) and tactical (plurality of protest repertoires through which resistance can be imagined and evaluated) (Tilly, 1978). These layers interact in the construction of political meaning: emotional orientations may activate particular representations of legitimacy, while representations of protest tactics provide concrete anchors through which emotions become politically actionable (Olumba, 2023).

Traditional collective action research identified emotions as central motivational drivers, with models evolving from discrete, unidimensional states towards recognizing emotional complexity and context‐dependence. The Social Identity Model of Collective Action (SIMCA; Van Zomeren et al., 2008) identified three core pathways: group‐based anger, collective efficacy and social identity, while Intergroup Emotions Theory (IET; Mackie et al., 2000) specified that outgroup‐directed anger generates approach‐oriented action tendencies. However, anger reliably predicts normative protest but not nonnormative or extreme actions (Tausch et al., 2011), suggesting it mobilizes within institutional frameworks rather than driving radicalization per se.

Researchers have since expanded attention beyond anger to other discrete emotions. Hope emerged as a critical sustaining force, operating sequentially with anger to link collective‐action frames to participation (Lizarazo Pereira et al., 2022; Wlodarczyk et al., 2017). Fear exhibits a dual character, both motivating vigilance and paralysing action (Kleres & Wettergren, 2017; Pearlman, 2013), while guilt and shame show target‐specific effects: anger towards outgroups predicted confrontational strategies, whereas shame predicted withdrawal (Iyer et al., 2007).

A critical theoretical refinement concerns emotion targets: participants experience more outgroup‐directed anger and contempt but also more self‐directed positive affect (Becker et al., 2011), and the same emotion can produce different behaviours depending on appraisals of control, responsibility and goal relevance (Lazarus, 1991), explaining why material damage (targeting systems) is psychologically differentiated from human harm (targeting persons), since the former aligns with systemic critique while the latter activates moral prohibitions against interpersonal violence.

Recent empirical work reveals that protest participants often report concurrent positive and negative feelings, and that mixed emotional states are common and consequential. Bettarelli et al. (2022) identified respondent classes characterized by high‐intensity anger and hope, finding that the combination is most strongly associated with non‐electoral protest and protest voting. Qualitative studies further document strategic blending of emotions: Kleres and Wettergren (2017) found that climate activists combined fear, hope, anger and guilt to manage fear and maintain morale. These findings challenge models that treat emotions as mutually exclusive and suggest that emotional plurality may enable activists to navigate the moral and strategic complexities of contentious politics.

Rather than viewing emotional contradictions as psychological inconsistency, affective polyphasia positions emotional plurality as a functional resource that enables individuals to navigate the moral ambiguity, strategic complexity and identity tensions inherent in contentious politics.

### The present study

Integrating Social Representations Theory with collective action research, this study examines how individuals evaluate political violence as a form of resistance, investigating: (1) the emotional plurality characterizing attitudes towards political violence; (2) the role of social representations in mediating the relationship between emotions and activism; (3) the moral boundaries differentiating acceptance of material versus human harm; and (4) the temporal polyphasia of anger (injustice‐oriented) and hope (efficacy‐oriented) as joint activators of political engagement.

## METHODOLOGY

### Participants and recruitment

A total of 444 participants (*N* = 444) were recruited through purposive convenience and snowball sampling using online platforms. Participants were required to be at least 18 years old and provide informed consent prior to completing the survey. Ages ranged from 18 to 87 years (*M* = 29.15, *SD* = 11.92, Mdn = 24). The sample was predominantly female (*n* = 262, 59.0%), followed by male participants (*n* = 156, 35.1%), non‐binary individuals (*n* = 19, 4.3%) and participants who preferred not to disclose their gender (*n* = 7, 1.6%).

Participants represented 64 countries across seven geopolitical regions: Eastern Europe (*n* = 166, 37.4%), the Middle East and North Africa (*n* = 85, 19.1%), Western countries (*n* = 70, 15.8%), East/Southeast Asia (*n* = 63, 14.2%), South Asia (*n* = 40, 9.0%), Sub‐Saharan Africa (*n* = 12, 2.7%) and Latin America (*n* = 8, 1.8%). This distribution is highly uneven, limiting the comparability of cross‐regional findings, which should be interpreted as sample‐specific rather than representative of broader geopolitical trends.

Political engagement varied: 41.0% reported no activism involvement, 38.1% occasional involvement, and 12.6% active participation. Politically, 45.0% identified with the left, 14.4% right, 12.4% centrist and 3.6% apolitical (24.3% declined to disclose); ideologies were most commonly democratic (47.3%), socialist/anti‐capitalist (17.1%), market‐liberal (10.8%) and national sovereignty (9.5%). Data were collected online between August and December 2025 via social media (X, Facebook, Instagram).

### Instrument design

The study utilized a mixed‐methods questionnaire titled Representations of Violence in Political Actions, developed in Google Forms. The instrument consisted of 48 items designed to capture the representational, emotional and moral dimensions of political violence through both structured Likert‐scale questions and open‐ended qualitative prompts.

### Measures

The questionnaire was divided into four primary conceptual profiles:

1. Legitimacy and Structural Violence (8 items): Items assessing representations of political violence (14 items) were written to operationalize key SRT constructs, structural violence, legitimacy and power asymmetry. Participants rated agreement with statements regarding the legitimacy of violence for social change, the relative harm of structural vs. physical violence, and the fairness of how marginalized groups' violence is portrayed (e.g. ‘The use of violence in resistance movements should be judged differently in colonized or oppressed contexts’).
2. Emotional Rationality and Motivations (10 items): assessed the ‘affective polyphasia’ of resistance, with items derived from the collective action literature (Mackie et al., 2000; Van Zomeren et al., 2008; Wlodarczyk et al., 2017). Participants rated the importance of emotional motivations behind violence and evaluated 10 emotions (e.g. anger, hope, grief, fear) as ‘rational and necessary’ in triggering movements against injustice.
3. Case‐Based Acceptability Scenarios (27 items): Participants rated the acceptability of specific historical and contemporary acts of resistance on a 7‐point scale from ‘Totally Unacceptable’ to ‘Totally Acceptable’. Scenarios were developed through iterative research group discussion and expert consensus, selected according to three criteria: (1) normative range, from widely accepted collective action (e.g. strikes, boycotts) to highly contested acts (e.g. hostage‐taking, armed resistance); (2) geographic and historical diversity, spanning anti‐colonial struggles (e.g. Mau Mau rebellion), contemporary liberation movements (e.g. Palestinian resistance) and Global North activism (e.g. climate protest); and (3) actor diversity, including armed movements, civil society organizations and individuals. Scenarios were written as brief neutral factual descriptions of actor, action and context. The resulting typology, general actions, specific liberation struggles, material damage, human confrontation and symbolic resistance, was derived empirically from participants' evaluations rather than imposed a priori. The instrument was reviewed internally prior to deployment; no formal external pilot was conducted, which we acknowledge as a limitation.
4. Qualitative Narratives: Participants responded to eight open‐ended questions; only responses to ‘Please state the top five emotions that come to your mind when you think of political activism that matters to you’ presented prior to the quantitative scales were analysed here.

## DESCRIPTIVE RESULTS

The analytical strategy follows a sequential, multi‐method logic moving from the pre‐categorical structure of emotional experience to its representational and behavioural consequences: etwork analysis captures spontaneous emotion co‐occurrence before analytical categories are imposed; exploratory factor analyses establish the latent structure of emotional repertoires, violence representations, and event evaluations; mediation analyses test how these structures relate to activism; and cluster analysis shows how they co‐occur within individuals and vary across contexts. Each step is necessary, the factor structures feed the mediation models and the mediation results ground the cluster profiles.

### Network analysis of emotions associated with political activism

First, we examined the spontaneous emotional associations participants reported in response to political activism prior to structured measurement, capturing the pre‐categorical organization of emotions.

Of the 444 participants, 433 (97.5%) provided valid responses (*M* = 4.20 emotions per participant, *SD* = 1.60, range: 1–12), with 63.5% listing exactly five emotions. After normalization of linguistic variants (e.g. anger/angry/rage to anger), the 1817 emotion mentions were reduced to 93 unique terms serving as network nodes. We note that some terms in the network (e.g. solidarity, justice, freedom) are value‐laden concepts rather than discrete emotion labels. Their inclusion was intentional: within an SRT framework, the co‐occurrence of emotions with moral‐political referents is itself a substantive finding, reflecting how participants frame their affective experience within broader social representations of violence and resistance (Jovchelovitch, 2008; Moscovici, 2008). These terms are best understood as anchoring nodes that reveal the normative architecture of the emotion network.

The most frequently mentioned emotions were anger (38.6%), hope (32.8%), and sadness (17.3%), followed by frustration (17.1%), determination (11.3%), pride (11.1%), fear (9.7%), anxiety (9.5%) and empathy (9.0%). The emotion frequency distribution was highly skewed: only 4 emotions were mentioned by more than 50 participants.

The co‐occurrence network consisted of 93 emotion nodes connected by 297 edges (minimum threshold: ≥2 co‐occurrences). Network density was 0.069, indicating a relatively sparse but structured network. The network was fully connected (1 component), suggesting that all emotions are linked through direct or indirect co‐occurrence pathways.

The anger–hope dyad emerged as the strongest connection in the network, with 62 co‐occurrences, followed by frustration–hope (42), anger–sadness (39), determination–hope (37) and anger–frustration (35). Notably, hope appeared in 3 of the top 5 strongest connections, underscoring its role as a central bridging emotion.

Anger exhibited the highest degree centrality (55 connections), followed closely by hope (52); PageRank showed anger (0.1181) and hope (0.1154) nearly equivalent in network influence, and betweenness centrality was highest for both (anger = 0.3414, hope = 0.3140), indicating these two emotions serve as critical pathways connecting disparate emotion profiles. Full network quality metrics are reported in Supplementary Note 2.

Louvain community detection identified 5 distinct emotion communities in the unweighted network:

1. Anxiety & Ambivalence Community (7 emotions): anxiety, enthusiasm, tiredness, confusion, admiration, concern, optimism. Characterized by mixed affective states and cognitive uncertainty.
2. Negative Affect & Intensity Community (25 emotions): anger, sadness, fear, disappointment, hopelessness, disgust, pain, depression, despair, rage and others. Represents intense negative emotions associated with injustice and distress, the entry point of political experience, signalling that something is wrong.
3. Hope & Action‐Oriented Community (24 emotions): hope, frustration, determination, empathy, solidarity, empowerment, compassion, courage, motivation and others. Reflects mobilization‐oriented emotions that bridge negative affect and collective action, signalling something can be done.
4. Pride & Positive Affect Community (18 emotions): pride, happiness, excitement, love, bravery, passion, joy, gratitude, strength and others. Encompasses positive emotions tied to collective identity and achievement.
5. Justice & Values Community (5 emotions): justice, freedom, equality, inclusion, human rights. Represents abstract moral and political values.

The emotional network reveals a structured affective system in which negative, action‐oriented, identity‐related, and moral‐value emotions are interconnected, with anger and hope functioning as the two dominant bridges enabling the transformation of distress into collective action, structural evidence that emotional plurality predicts non‐electoral protest more effectively than single emotions (Bettarelli et al., 2022) (Figure 1).

**FIGURE 1:**
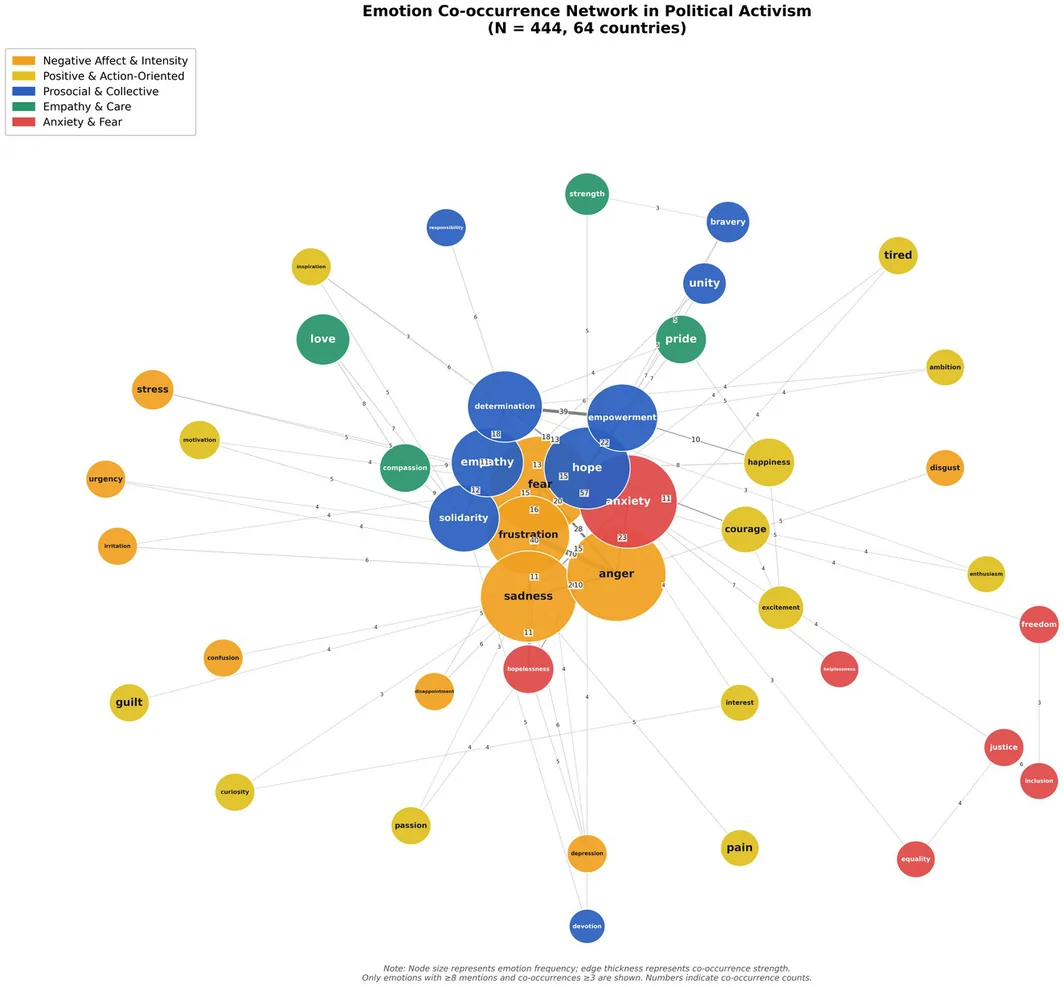
Co‐occurrence network of emotions associated with political activism.

### Violence representations

An exploratory factor analysis was conducted on 14 items relating to representations of political violence (*N* = 444), which demonstrated good suitability (KMO = 0.86; Bartlett's test: *χ* ^2^(91) = 1736.00, *p* < .001; maximum likelihood extraction, oblimin rotation). A three‐factor solution was identified, accounting for 41.9% of the total variance, with excellent fit (RMSEA = 0.046, 90% CI [0.032, 0.060]; TLI = 0.948; *χ* ^2^(52) = 101.00, *p* < .001), preferred over two‐ and four‐factor alternatives on the basis of parallel analysis, scree plot inspection and the Bayesian Information Criterion (BIC = −216).

Factor 1: Power‐Critical Representations (5 items, 18.1% variance, *α* = .78) was defined by items critiquing the state's monopoly on violence and unfair portrayals of marginalized groups, including items on structural violence and the necessity of violence for agency in oppressed contexts.

Factor 2: Moral Values (5 items, 13.8% variance, *α* = .74) focused on the moral and value‐based grounding of justice (e.g. justice as values; political ethics; moral justification).

Factor 3: Violence as a Tool (4 items, 10.0% variance, *α* = .45) comprised items referring to the instrumental use of violence as a political tactic, with symbolic/cultural resistance loading negatively. This low internal consistency reflects the construct's inherent heterogeneity, items span a range of tactical positions, and the negative loading of symbolic resistance suggests a perceived trade‐off between physical and symbolic action rather than a unified attitude, so this factor is retained for descriptive purposes only and excluded from all inferential analyses (correlation network, mediation, cluster analysis), its value lying in documenting that participants distinguish moral‐ethical from tactical‐instrumental framings of violence.

Inter‐factor correlations revealed that Power‐Critical Representations correlated strongly with Moral Values (*r* = .41), but Violence as a Tool was unrelated to Moral Values (*r* = .01) while moderately associated with Power‐Critical Representations (*r* = .32), suggesting the instrumental framing of violence is driven not by the strength of ethical commitments but by whether one's representational framework encodes power asymmetry and structural delegitimization of resistance, a distinction we return to in the Discussion.

### Emotional rationality

An exploratory factor analysis examined the structure of emotional responses to political activism (*N* = 444; KMO = 0.86; Bartlett's test: *χ* ^2^(45) = 1824.00, *p* < .001). A three‐factor solution explaining 56.0% of the variance was supported by scree plot, parallel analysis and the Bayesian Information Criterion (BIC = −59.2; RMSEA = 0.064, 90% CI [0.044, 0.085]; TLI = 0.954):

Factor 1: Transformative Emotions (4 items, 22.2% variance, *α* = .80): anger, frustration, grief and hope.

Factor 2: Self‐evaluative Emotions (4 items, 18.7% variance, *α* = .79): guilt, shame, anxiety and pride.

Factor 3: Threat Emotions (2 items, 15.1% variance, *α* = .79): fear and hopelessness.

As expected with oblique rotation, the factors were moderately correlated: Transformative and Threat emotions (*r* = .60), Self‐evaluative and Threat (*r* = .52), and Transformative and Self‐evaluative (*r* = .40). These correlations suggest emotional responses to injustice coexist within the same representational field rather than being oppositional, an affective polyphasia in which fear and hope are simultaneously present, and resistance emerges not from the absence of fear but its transformation into action‐oriented emotions (Bou Zeineddine & Vollhardt, 2024; Fanon, 1967).

Factor labels reflect appraisal theory and each cluster's functional role in collective action. Transformative emotions (anger, frustration, grief, hope) share a backward‐looking moral attribution combined with a forward‐looking mobilizing orientation; grief was included because in political contexts it functions as a politically directed recognition of loss that anchors moral commitment to change (Butler, 2004), rather than passive mourning. Self‐evaluative emotions (guilt, shame, anxiety, pride) are united by an internal responsibility‐attribution and agentic control appraisal; their primary object is the self rather than an external agent (Tracy & Robins, 2004; Wohl et al., 2006), and can be experienced individually or collectively. Threat emotions (fear, hopelessness) share a threat‐appraisal structure of external danger and uncontrollability (Kleres & Wettergren, 2017; Lazarus, 1991); fear was placed here rather than with self‐evaluative emotions because its co‐loading with hopelessness reflects this shared external orientation. This combination is consistent with the political emotion qahr, documented among oppressed Arab communities, capturing crushing powerlessness under structural domination that is both individually felt and socially shared (Obeid, 2023).

### Contentious political actions

An exploratory factor analysis was conducted on 27 items representing diverse historical and contemporary acts of political resistance (*N* = 444; KMO = 0.95; Bartlett's test: *χ* ^2^(325) = 6592.00, *p* < .001), with the factor structure not specified a priori, consistent with a Social Representations Theory approach in which latent dimensions emerge from participants' own evaluative patterns rather than a predetermined taxonomy. A five‐factor solution was identified, accounting for 55.2% of the variance (RMSEA = 0.059, 90% CI [0.053, 0.065]; TLI = .919; *χ* ^2^(205) = 521.00, *p* < .001), preferred over four‐ and six‐factor alternatives on the basis of parallel analysis, scree plot inspection and the Bayesian Information Criterion (BIC = −729).

Factor 1: General Actions (6 items, 13.0% variance, *α* = .83): normative or non‐violent protest tactics including boycotts, strikes, occupations and graffiti.

Factor 2: Specific Resistance (7 items, 14.1% variance, *α* = .88): historically and contextually situated struggles for liberation, including the Zapatista uprising, Palestinian resistance and the Mau Mau rebellion.

Factor 3: Material Damage (4 items, 13.3% variance, *α* = .82): disruption of infrastructure, including blocking transport and rioting against regimes.

Factor 4: Human Damage (6 items, 11.7% variance, *α* = .82): the most radical forms of confrontation involving direct harm to persons, including kidnapping and hostage‐taking.

Factor 5: Symbolic Damage (2 items, 3.1% variance, *α* = .61): destruction of national or cultural symbols, including vandalizing artwork and toppling statues.

Inter‐factor correlations indicated a partial resistance continuum of escalating physical harm: General Actions (*r* = .63 with Specific Resistance), Material Damage (*r* = .55) and Human Damage (*r* = .56) form a gradient from normative to radical tactics. Specific Resistance, however, occupies a structurally distinct position: despite involving historically violent acts, it was rated with high acceptability and low variance, comparable to General Actions, while correlating significantly with both damage factors, suggesting it is evaluated not by harm level but by historical and geopolitical context, since acts anchored in recognized liberation struggles acquire a distinct moral status through social anchoring (Moscovici, 2008).

A mean–variance analysis of all 27 scenario items (Figure 2) distinguished acceptability (mean) from social contestation (variance), with the variance axis divided at the median (Mdn = 3.60). Quadrant labels reflect direction of evaluation (acceptance vs. rejection) and degree of agreement (consensus vs. contested), where ‘contested’ denotes wide disagreement rather than a necessarily bimodal distribution. General Actions and Specific Resistance items showed predominantly high acceptability and below‐median variance, indicating broad consensus; Human Damage items displayed low acceptability and high variance, marking sites of moral conflict; and Symbolic Damage items showed consistently high variance regardless of mean acceptability, suggesting symbolic iconoclasm is a highly contested domain.

**FIGURE 2:**
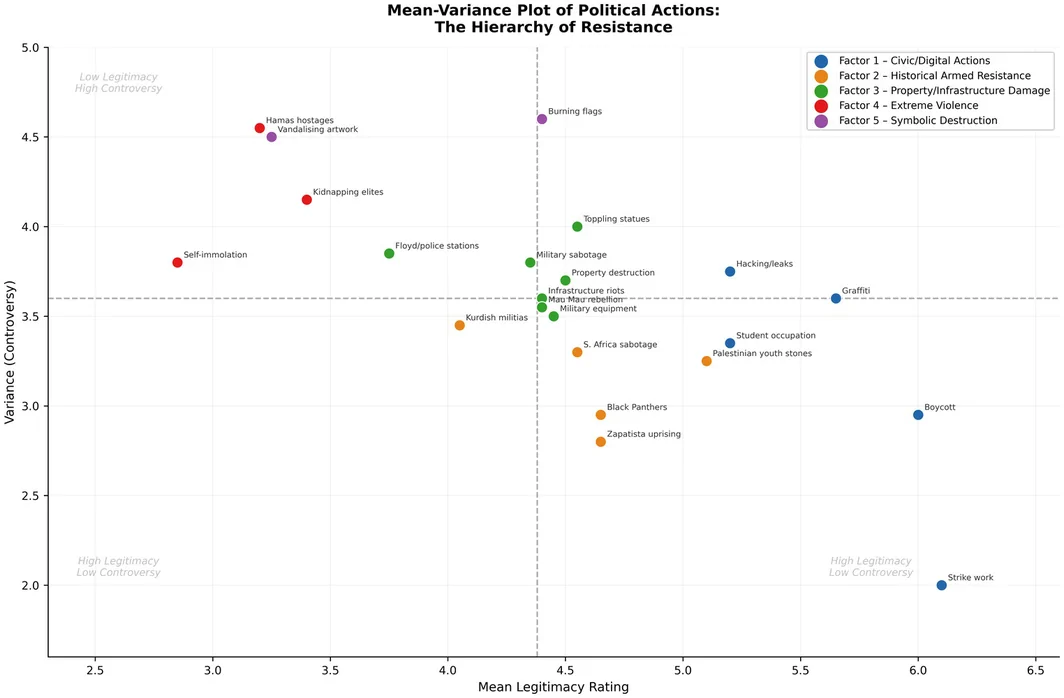
Mean–variance distribution of political resistance scenarios revealing consensus and contestation.

### Correlation matrix between factors

To examine how emotional responses, violence representations and judgements of contentious actions interconnect, we conducted correlation analyses across all factors, excluding the low‐reliability ‘Violence as a Tool’ factor (*α* = .45). Figure 3 visualizes these relationships as a force‐directed network, where spatial proximity reflects correlation strength and only inter‐domain correlations (*r* > .15) are displayed.

**FIGURE 3:**
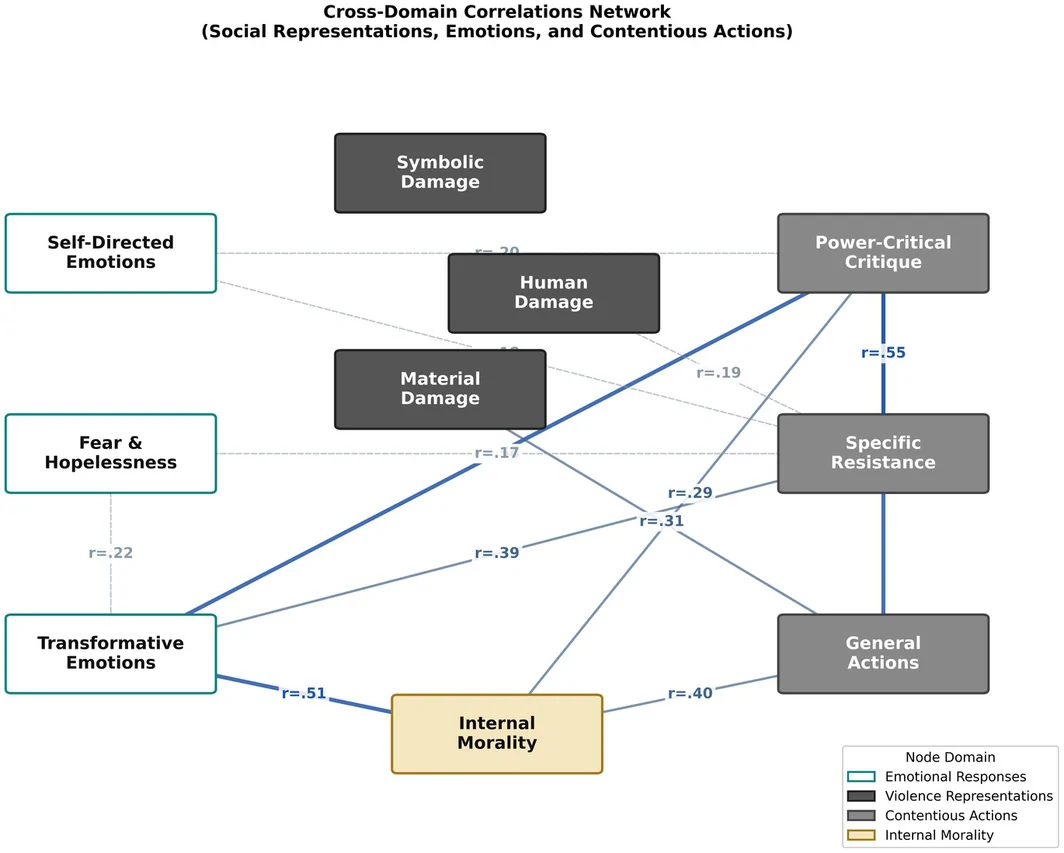
Integrative network of political resistance: cross‐domain correlations.

The ‘Activist Core’: Power‐Critical Representations emerged as a central psychological hub, correlating strongly with General Actions (*r* = .61), Specific Resistance (*r* = .56), and Transformative Emotions (*r* = .54), indicating that anger, frustration, grief and hope are cognitively anchored in structural critiques of power, forming an ‘Activist Core’ visible as a spatial cluster in Figure 3.

The Decoupling of Internal Morality and Extreme Tactics: Moral Values showed moderate correlations with General Actions (*r* = .41) and Specific Resistance (*r* = .30) but near‐zero correlations with Material Damage (*r* = −.00) and Human Damage (*r* = .02), revealing that individuals who strongly ground justice in moral values are no more or less likely to support infrastructure sabotage or hostage‐taking.

Symbolic Damage as a Representational Outlier: Symbolic Damage remained isolated in the network, showing no significant correlations with General Actions (*r* = .02), Material Damage (*r* = .07) or Transformative Emotions (*r* = −.04), suggesting symbolic acts are evaluated through a fundamentally different moral framework.

### Mediation analysis: Event evaluations as mediators between emotional dispositions and political activism

To examine whether event evaluations mediate the relationship between emotional dispositions and activism (after multiple linear regression analysis in Supplementary Note 3), we conducted three parallel mediation analyses using bootstrap methods with 5000 iterations and bias‐corrected 95% confidence intervals, with the three emotion factor scores as predictors, the five event evaluation factors as parallel mediators and self‐reported activism (1–7 ordinal scale) as the outcome. Given the cross‐sectional design, all mediation findings reflect statistical associations and indirect paths rather than causal processes; the language of ‘pathways’ and ‘indirect effects’ is used in the standard sense and does not imply temporal or causal ordering.

The mediation model tested here reflects an analytical ordering grounded in Social Representations Theory. Following Marková (2003), injustice becomes meaningful through the representational process itself, a dialogical encounter in which emotions anchor the object as politically significant, operating at a more immediate level than evaluating specific strategies, which requires further cognitive elaboration (Jovchelovitch, 2008; Wagner, 1998). The questionnaire mirrored this process, presenting emotion items before scenario evaluations. This ordering is not a causal claim but an analytical representation of how the representational process unfolds; cross‐sectional data preclude causal inference, and the model captures relational structure rather than temporal precedence.

#### Mediation Model 1: Transformative emotions and political activism

Transformative emotions demonstrated a significant positive total effect on Activism (*c* = 0.107, *ρ* = .097, *p* = .042). The total indirect effect through all five event evaluations was significant (Σ*ab* = 0.194, 95% CI [0.099, 0.287]), indicating an indirect‐only effect with suppression (proportion mediated: 181%). Three significant specific indirect pathways emerged: (1) through General Actions (*ab* = 0.069, 95% CI [0.003, 0.146]), (2) through Material Damage (*ab* = 0.050, 95% CI [0.013, 0.094]) and (3) through Human Damage (*ab* = 0.028, 95% CI [0.004, 0.061]). The direct effect became non‐significant and negative (c′ = −0.087), consistent with inconsistent mediation.

This pattern suggests that transformative emotions do not translate directly into political engagement; they become mobilizing only when anchored in specific representations of protest tactics, with specific forms of action emerging as the significant indirect paths in this cross‐sectional sample (Figure 4).

**FIGURE 4:**
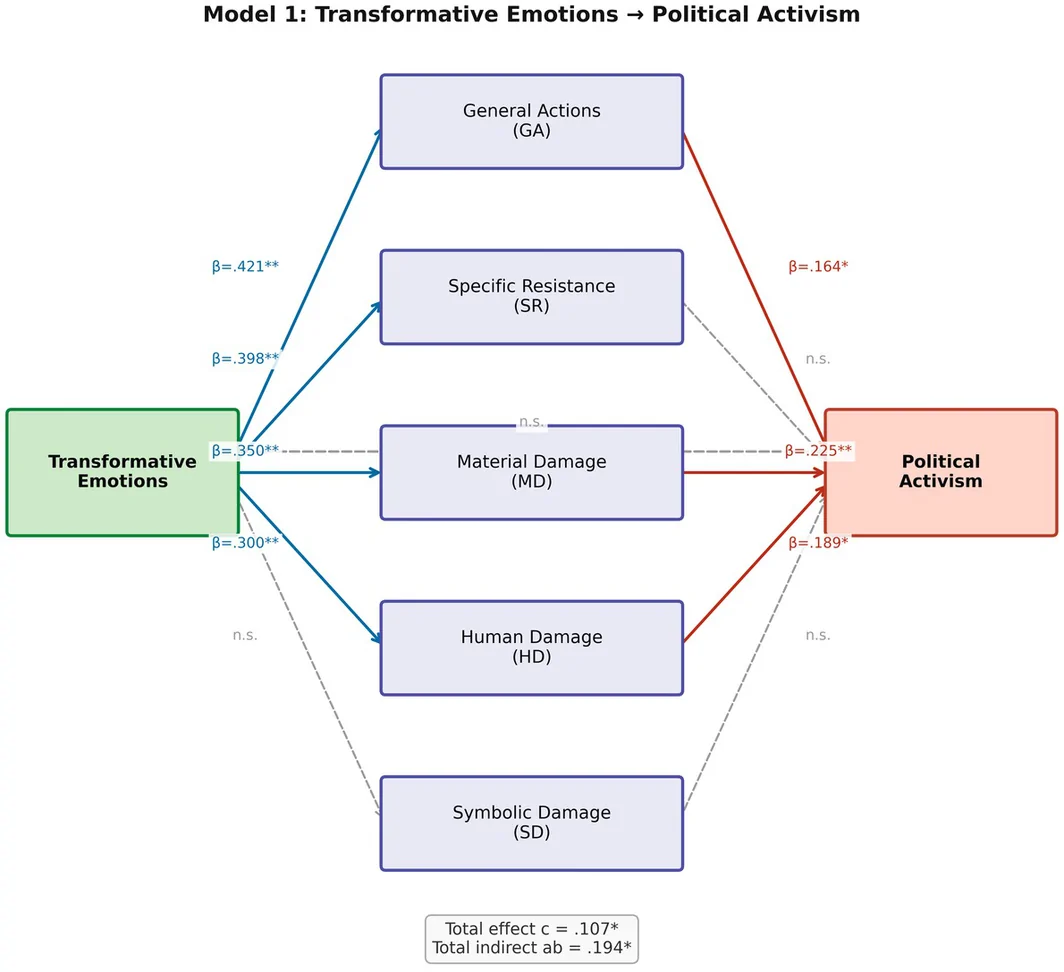
Parallel mediation model testing event evaluations as mediators between transformative emotions and political activism.

#### Mediation Model 2: Self‐directed emotions and political activism

Self‐evaluative emotions showed a significant positive total effect on Activism (*c* = 0.157, *ρ* = .114, *p* = .016). The total indirect effect was significant (Σ*ab* = 0.125, 95% CI [0.059, 0.203]), accounting for 80% of the total effect. Three significant specific indirect pathways emerged: (1) through Material Damage (*ab* = 0.029, 95% CI [0.003, 0.066]), (2) through Human Damage (*ab* = 0.024, 95% CI [0.000, 0.059]) and (3) through Symbolic Damage (*ab* = 0.039, 95% CI [0.011, 0.076]). Notably, self‐evaluative emotions uniquely predicted Symbolic Damage evaluations (*a* = 0.217). The direct effect remained positive but non‐significant (*c*′ = 0.032), indicating substantial but not complete mediation.

This pattern suggests that self‐evaluative emotions function as normative evaluations of legitimate motivation for collective action, with their unique association with symbolic actions indicating they are especially linked to forms of resistance that express moral positioning rather than intervening in material structures (Figure 5).

**FIGURE 5:**
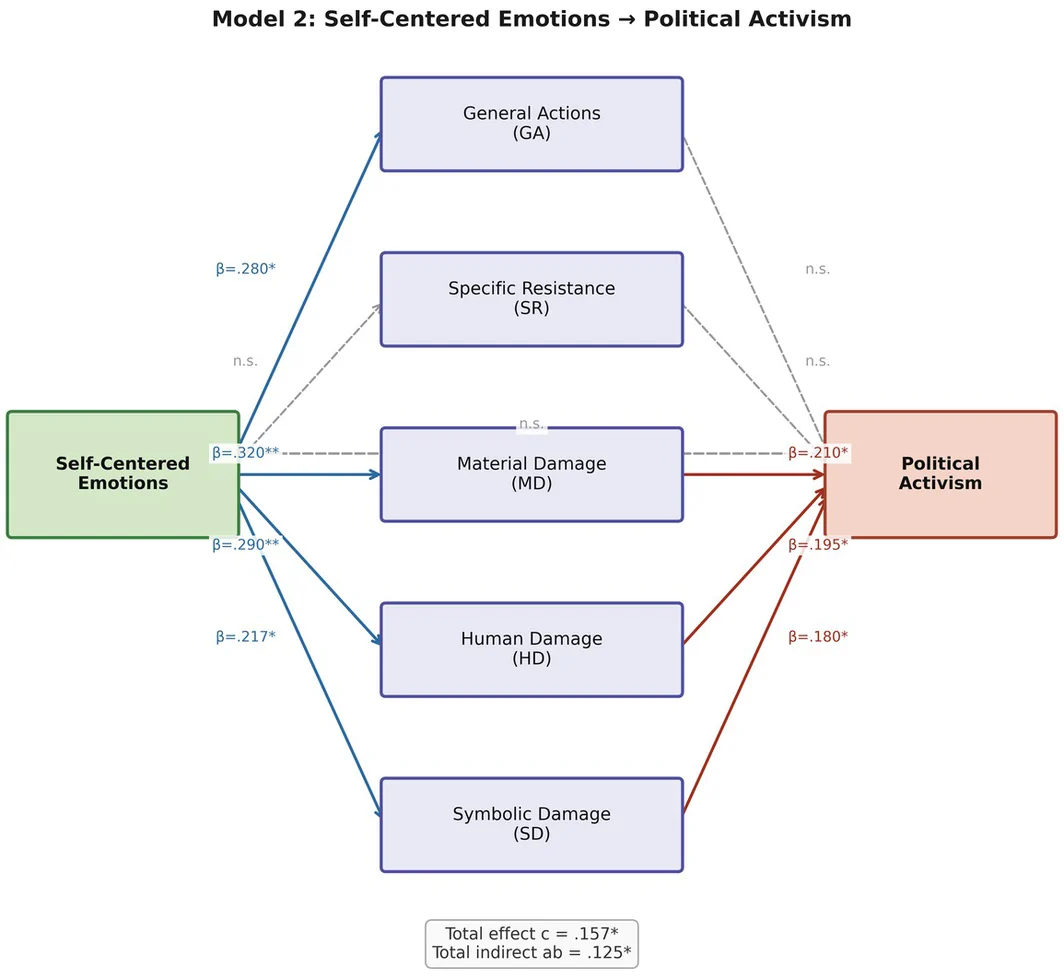
Parallel mediation model testing event evaluations as mediators between self‐evaluative emotions and political activism.

#### Mediation Model 3: Threat emotions and political activism

Threat emotions demonstrated a non‐significant negative total effect on Activism (*c* = −0.087, *ρ* = −.038, *p* = .421), yet the total indirect effect through event evaluations was significant and positive (Σ*ab* = 0.095, 95% CI [0.027, 0.165]). This inconsistent mediation (suppression) indicates that event evaluations convey a positive indirect effect masked by a stronger negative direct effect. Two specific pathways were significant: (1) through General Actions (*ab* = 0.038, 95% CI [0.002, 0.083]) and (2) through Material Damage (*ab* = 0.030, 95% CI [0.004, 0.066]). The proportion mediated was −109%.

This pattern suggests that threat emotions are inherently ambivalent: they may directly inhibit action through avoidance or risk aversion, yet indirectly facilitate engagement when protest tactics are interpreted as legitimate, becoming mobilizing only through their anchoring in representations of resistance (Figure 6).

**FIGURE 6:**
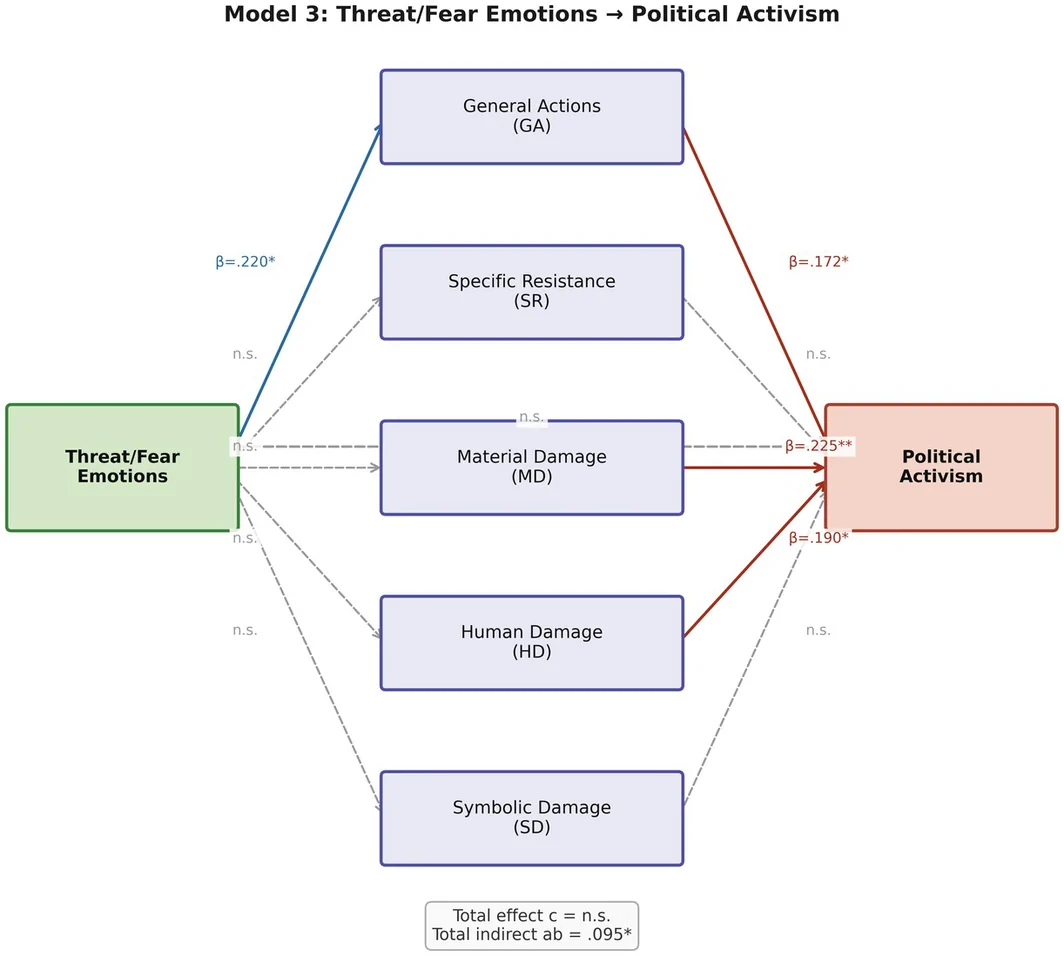
Parallel mediation model testing event evaluations as mediators between threat emotions and political activism.

Across the three models, event evaluations consistently mediated the relationship between emotional dispositions and activism, though the strength and form varied by type: transformative emotions operated primarily through event evaluations, self‐evaluative emotions showed both indirect and residual direct effects, and threat emotions exhibited an inconsistent pattern of opposing direct and indirect influences.

### Profile analysis

To complement the variable‐centred analyses and capture how emotional evaluations co‐occur within individuals, we conducted a profile analysis based on the three emotion factors. These profiles are not fixed types but structured constellations of co‐existing, potentially contradictory repertoires reflecting affective polyphasia in relation to political action.

A *K*‐means cluster analysis was conducted on the three emotion factors (standardized variables, *M* = 0, *SD* = 1). Solutions ranging from *k* = 2 to *k* = 6 were evaluated using the Silhouette coefficient, Calinski–Harabasz index and Davies–Bouldin index. The five‐cluster solution was retained as optimal (Davies–Bouldin = 1.054; Silhouette = 0.306; Calinski–Harabasz = 223.2). One‐way ANOVAs confirmed that all three emotion factors significantly differentiated the clusters: Transformative Emotions, *F*(4, 439) = 253.34, *p* < .001, *η* ^2^ = 0.698; Self‐evaluative Emotions, *F*(4, 439) = 182.50, *p* < .001, *η* ^2^ = 0.624; Threat, *F*(4, 439) = 242.89, *p* < .001, *η* ^2^ = 0.689 (Figure 7).

**FIGURE 7:**
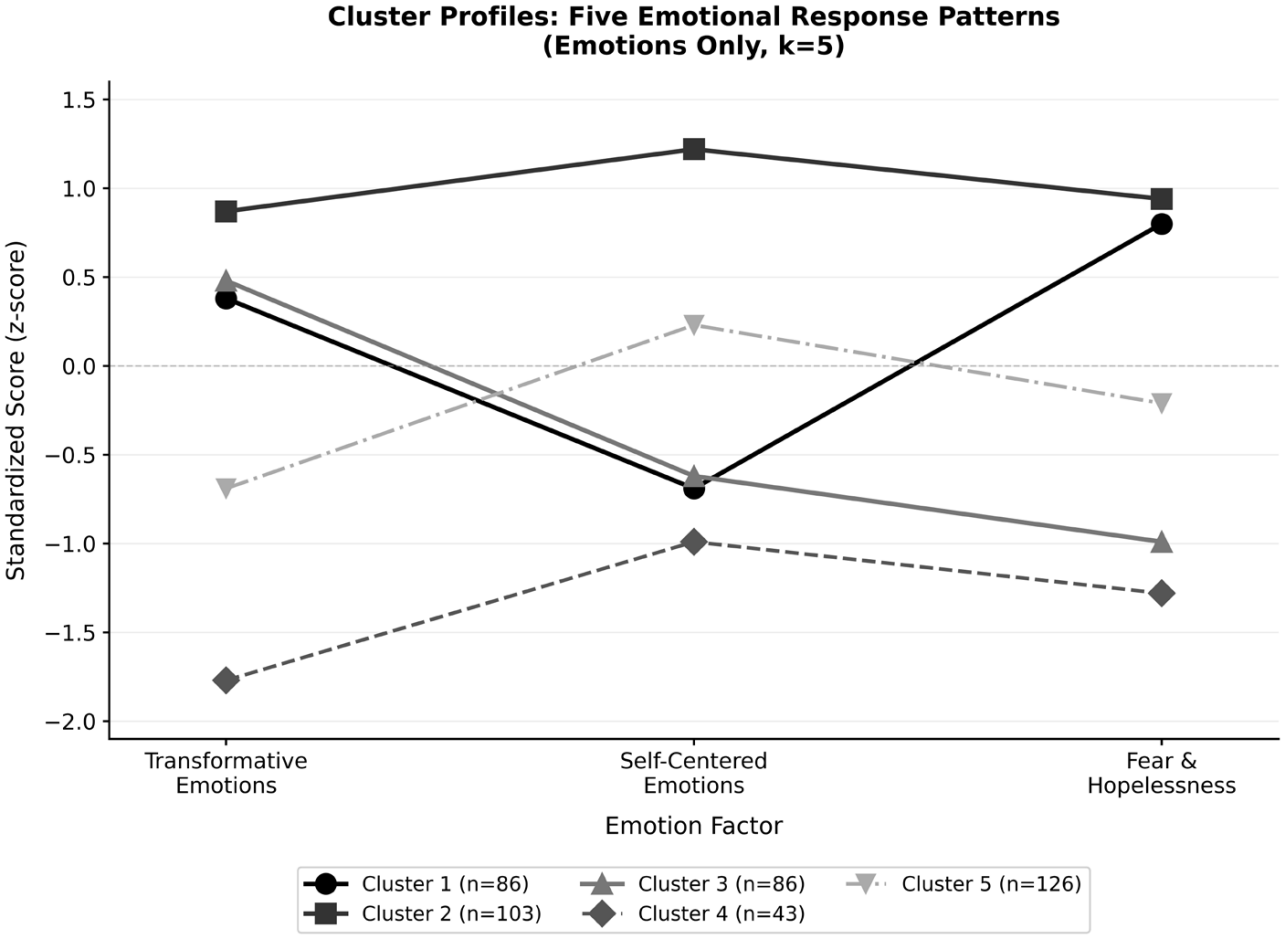
Standardized scores of five emotion‐based cluster profiles across three emotional response factors.

Profile 1: Crisis‐Driven Mobilization (*n* = 86, 19.4%) combined moderate transformative emotions with low self‐evaluative emotions and high threat, suggesting an orientation towards action grounded in perceived external threat rather than moral self‐evaluation.

Profile 2: Polyphasic Responders (*n* = 103, 23.2%) exhibited high endorsement across all three emotion factors, reflecting an inclusive orientation in which multiple emotional pathways to activism are recognized as valid.

Profile 3: Empowered Resisters (*n* = 86, 19.4%) combined moderate transformative emotions with low self‐evaluative emotions and very low threat, reflecting an orientation towards action grounded in agency rather than fear.

Profile 4: Disengaged Sceptics (*n* = 43, 9.7%) showed uniformly low endorsement across all factors, suggesting a general rejection of emotionally‐driven activism.

Profile 5: Cautious Conformists (*n* = 126, 28.4%) combined low transformative emotions with moderate self‐evaluative emotions and low threat, reflecting caution grounded in moral evaluation rather than emotional escalation.

These profiles are consistent with emerging research identifying distinct subpopulations of political engagement, where social class, ideological identification and movement identity interact to produce qualitatively different trajectories of collective action (Álvarez et al., 2024).

Clusters differed significantly across all three violence representation dimensions (full statistics in Tables S2 and S3). Power‐Critical Representations showed the largest effect, with Polyphasic Responders and Crisis‐Driven Mobilizers scoring highest and Disengaged Sceptics lowest; Moral Values showed a similar pattern, again highest among Polyphasic Responders and lowest among Disengaged Sceptics.

Emotional response profiles also differed significantly across all five action dimensions (Tables S2 and S3). Polyphasic Responders and Crisis‐Driven Mobilizers scored highest on General Actions, with Disengaged Sceptics and Cautious Conformists lowest. Notably, Polyphasic Responders were the only profile with positive endorsement of Human Damage (*M* = 0.28), while all others scored at or near zero, and Cautious Conformists uniquely scored highest on Symbolic Damage.

Significant differences emerged for political activism (*p* < .001), geopolitical region (*p* = .014) and political ideology (*p* = .046), while age, gender, political orientation and religion showed no significant differences. Given the uneven regional distribution of the sample and the absence of measurement invariance testing across regions, all cross‐regional comparisons reported below should be treated as exploratory and sample‐specific.

Polyphasic Responders reported the highest activism levels (Mdn = 4.0, *M* = 3.39), while Crisis‐Driven Mobilizers reported the lowest (Mdn = 2.0, *M* = 2.40).

Geopolitical region was significantly associated with emotional response profiles, *χ* ^2^(24) = 41.63, *p* = .014, Cramér's *V* = .153: Eastern Europe, the largest regional group (37.4%), showed relatively higher representation in Crisis‐Driven Mobilizers and Cautious Conformists, while Disengaged Sceptics were relatively more frequent in East/Southeast Asia. Given the uneven regional distribution of the sample and the small effect size (Cramér's *V* = .153), these associations should be interpreted as exploratory patterns within this convenience sample, not as indicators of broader geopolitical trends (Figure 8).

**FIGURE 8:**
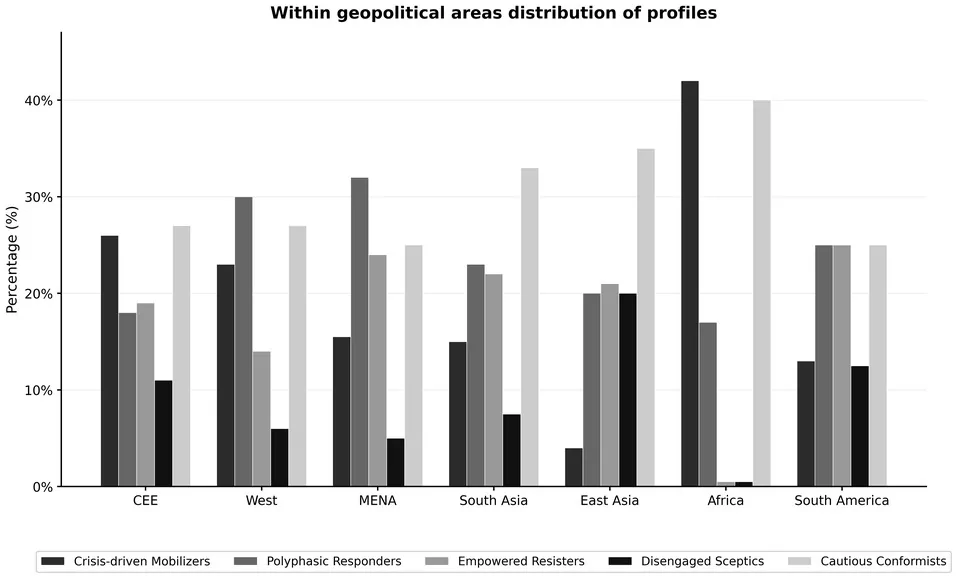
Within‐region distribution of emotional response profiles.

Political ideology showed a marginally significant association, *χ* ^2^(16) = 26.61, *p* = .046, Cramér's *V* = 0.122, with mobilizing profiles (Polyphasic Responders and Empowered Resisters) more prevalent among Socialist/Communist respondents, and Cautious Conformists more prominent among sovereigntist groups (Figure 9).

**FIGURE 9:**
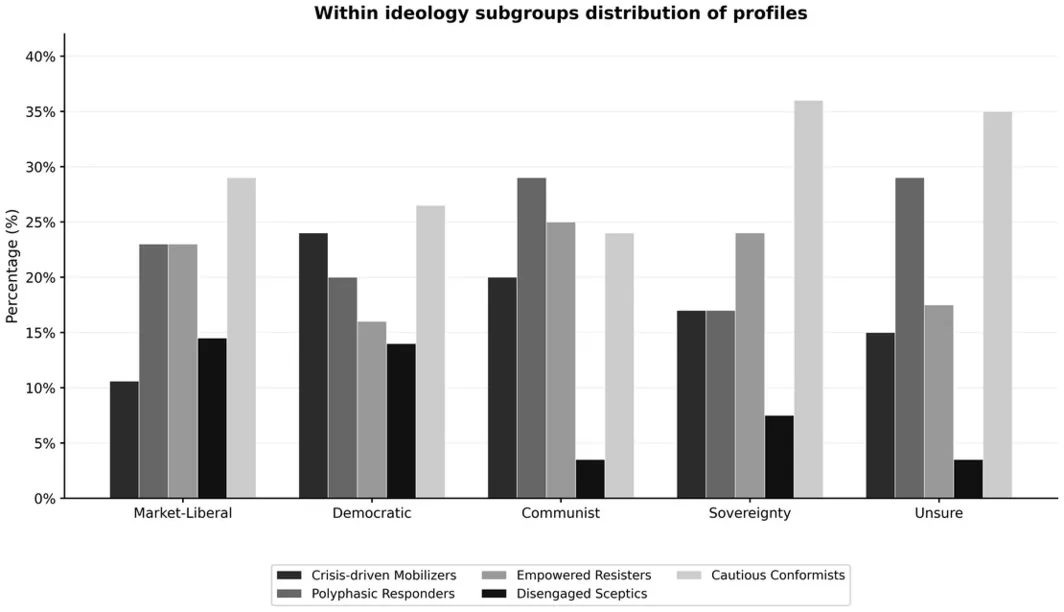
Within‐ideology distribution of emotional response profiles.

Profile labels were assigned based on the convergent pattern of emotion factor scores, representational framing, resistance endorsement, and activism across each cluster (see Table 1). Polyphasic Responders (23.2%) are the most emotionally inclusive and politically engaged profile, high across all three emotional factors and uniquely tolerant of the full spectrum of resistance tactics, representing a form of affective polyphasia in which contradictory emotional rationalities coexist as a resource for navigating moral complexity. Disengaged Sceptics (9.7%) show uniformly low endorsement across all dimensions; ‘sceptics’ captures not mere apathy but an absence of the structural critique and moral framing through which violence becomes politically intelligible. Crisis‐Driven Mobilizers (19.4%) are propelled by threat and transformative emotions while rejecting self‐evaluative affect: ‘crisis‐driven’ reflects injustice constituted as an acute, threatening situation, while ‘mobilizers’ designates emotional readiness for action that remains constrained by moral limits rather than expressed in reported activism. Empowered Resisters (19.4%) are anchored in transformative emotions alone; it is precisely the absence of self‐evaluative and threat emotions that constitutes the ‘empowerment’, enabling morally confident engagement without internal conflict. Cautious Conformists (28.4%), the largest group, show moral discomfort without the mobilizing force of anger or hope; ‘conformists’ designates not passive compliance but below‐average structural critique, consistent with normative frameworks that tolerate expressive but not instrumental transgression.

**TABLE 1:** Participant profiles identified by *K*‐means cluster analysis on the three emotion factors and significant differences among profiles between other variables.

		Crisis‐driven mobilizers	Polyphasic responders	Empowered resisters	Disengaged sceptics	Cautious conformists
Emotional factors	Transformative	High			Low	Medium
	Self‐evaluative	Low	High	Low		Medium
	Threat	High		Low		Medium
Sample (*N* = 444)		*n* = 86 19.4%	*n* = 103 23.2%	*n* = 86 19.4%	*n* = 43 9.7%	*n* = 126 28.4%
Violence factors	Power‐critical	High		Medium	Low	Medium
	Moral values	High		High	Low	Medium
	Violence as a Tool	Medium	High	Medium	Low	Medium
Acceptability of political events and actions	General actions	High		Medium	Low	
	Specific resistance	Medium	High	Medium	Low	Medium
	Material damage	Medium	High	Medium	Low	Medium
	Human damage	Low	Medium‐High	Low		
	Symbolic damage	Low	Medium			High
Significant independent variables	Activism	Low	High		Low‐medium	Low
	Geopolitical region	CEE, Sub‐Saharan Africa	Western Europe, MENA, South America	MENA, South America, South Asia	East Asia, South America	CEE, Western Europe, MENA, South Asia, East Asia, Sub‐Saharan Africa, South America
	Political ideology	Democratic and Communist	Communist and Unsure	Communist and Sovereignty	Market‐Liberal and Democratic	Market‐Liberal, Democratic, Sovereignty and Unsure

Geopolitical and ideological patterns further underscore that these profiles are not psychologically universal: Crisis‐Driven and Polyphasic profiles were more frequent among participants from contexts historically marked by colonial domination and authoritarian rule (CEE, MENA), though small cell sizes for Sub‐Saharan Africa (*n* = 12) and Latin America (*n* = 8) preclude generalization, while Cautious Conformists dominated across a wider range of regions and ideologies, suggesting that moral caution without mobilization may be the modal response in relatively stable or institutionally constrained contexts. Overall, these findings demonstrate that the legitimacy of political violence is negotiated through polyphasic systems in which emotional, representational and tactical evaluations are jointly configured.

## DISCUSSION

This study examined how individuals negotiate the legitimacy of political violence as resistance through the lens of Social Representations Theory (SRT), with particular attention to the emotional and cognitive structures that shape moral evaluations of protest tactics. Drawing on data from 444 participants across 64 countries, we analysed the interplay between emotional repertoires, evaluations of protest legitimacy and support for activism. Our findings challenge linear emotion‐to‐action models and reveal instead a complex, polyphasic system in which emotions and cognitions operate through multiple, sometimes contradictory pathways.

Three core empirical contributions emerge, elaborated below: network and factor analyses identified anger and hope as central, co‐occurring emotions with complementary temporal orientations; mediation analyses identified three distinct emotion‐specific indirect pathways associated with activism; and cluster analysis identified five emotional profiles varying systematically by geopolitical context and ideological orientation.

These findings extend Social Representations Theory and refine collective action models, as detailed in the Theoretical Contributions section below.

### The anger‐hope dyad as affective polyphasia

Network analysis revealed anger as the emotion with the highest degree centrality, while hope exhibited the highest betweenness centrality, functioning as a bridge between emotional clusters. These two emotions co‐occurred frequently rather than operating as opposing forces, challenging conventional wisdom that positions anger and hope as incompatible or sequential in collective action (Van Zomeren et al., 2008); instead, they constitute a polyphasic affective structure with distinct but complementary temporal orientations.

Anger, as a backward‐looking emotion, is fundamentally attributional: it directs attention to injustice, identifies responsible agents and generates moral outrage (Mackie et al., 2000; Srinivasan, 2018). Hope, conversely, is forward‐looking and efficacy‐focused, orienting actors towards possible futures and sustaining engagement when immediate outcomes remain uncertain (Wlodarczyk et al., 2017); it operates through perceived efficacy, the belief that collective action can produce meaningful change, making it a distinct motivational force that predicts support for social change over and above other emotions (Greenaway et al., 2016). This integration is not a resolution of contradiction but a functional coexistence of multiple rationalities, consistent with Moscovici's (2008) conceptualization of cognitive polyphasia as a resource rather than a deficit, and with a systems view of political emotion in which discrete emotions function as interdependent activators within broader socio‐political systems (Leach et al., 2024; Leach & Bou Zeineddine, 2021).

Hope's bridging function is particularly significant: it channels distress towards mobilization, linking present grievances to imagined alternatives (Reed, 2004), and its high betweenness centrality indicates that it mediates connections between otherwise disparate emotional experiences, sustaining engagement across shifting political contexts, consistent with emotions functioning as accelerators of motivational forces across stages of collective action (Van Troost et al., 2013). This coexistence of anger and hope enables what Fanon (1967) described as the transformative potential of resistance: a simultaneous rejection of oppressive structures and affirmation of alternative futures.

The emergence of a discrete Justice & Values community, comprising justice, freedom, equality and inclusion, warrants brief interpretive attention. Though not emotion terms per se, their emergence as a distinct co‐occurrence cluster is consistent with SRT: participants' emotional responses to political violence are anchored in explicit moral‐political frameworks, with the co‐occurrence of these value terms alongside anger and pride suggesting that emotional reactions are normatively structured, grounded in beliefs about what is just, equal and free (cf. Leach et al., 2024). This aligns with the broader SRT argument that social representations function as cognitive‐affective schemata that render social phenomena meaningful, and carries normative implications from a critical psychology perspective: understanding the emotional architecture of resistance can inform practices of liberatory psychology and community empowerment (Fox et al., 2009).

### Emotion‐specific pathways to activism: Three routes

The mediation analyses revealed a key finding: emotions were associated with activism through three distinct pathways that vary by emotion type, challenging models that treat emotions as uniform motivational forces. Transformative emotions showed full mediation, operating indirectly through evaluations of general actions, material damage and human harm, indicating that such emotions were associated with action primarily when anchored in shared representations of protest legitimacy, requiring objectification within socially constructed meanings of justified resistance (Moscovici, 2008). This supports arguments that emotions become politically consequential through shared moral frameworks rather than raw intensity (Becker & Tausch, 2017), with cognitive evaluations serving as the significant indirect paths through which emotions were associated with action in this sample, consistent with appraisal theories (Smith & Ellsworth, 1985; Van Troost et al., 2013).

The null direct effect of transformative emotions on activism diverges from SIMCA predictions (Van Zomeren et al., 2004, 2008): we interpret this as a boundary condition specific to the political violence context, where support for resistance violence requires an additional cognitive step, evaluating whether violence is legitimate, before emotions translate into behavioural engagement, a mechanism specified by the full mediation found here but absent from prior models.

The failure of Specific Resistance to predict activism reflects a principle‐implementation gap: moral endorsement of historically situated liberation struggles does not translate into personal mobilization, as participants likely evaluate these acts as legitimate for others rather than personally actionable, whereas General Events predicts activism because these are actions participants can envisage performing themselves.

Self‐evaluative emotions showed partial mediation, retaining direct effects and uniquely predicting symbolic damage. This suggests additional identity‐based mechanisms linked to moral self‐evaluation (Van Zomeren et al., 2012) and aligns with decolonial perspectives emphasizing epistemic resistance (Mignolo, 2007; Smith, 2019; Tuck & Yang, 2012).

Threat emotions exhibited suppression effects: while directly inhibiting action, they indirectly promote engagement when framed as legitimate responses to danger, reflecting their ambivalence (Fanon, 1967). The near‐significant positive association between self‐evaluative emotions and activism diverges from individual‐level findings that guilt and shame are poor action motivators (Shepherd et al., 2011). We suggest this reflects collective rather than individual moral psychology: collective guilt about structural complicity and pride in resistance identity can motivate reparative political engagement (Wohl et al., 2006), consistent with the unique role of self‐evaluative emotions in predicting symbolic resistance actions found in our mediation analyses.

The threat emotions cluster, combining fear and hopelessness, resonates with Obeid's (2023) concept of qahr, a political emotion documented among Arab communities under domination that integrates fear of power with hopelessness about change into a socially shared affective state, supporting the interpretation of threat emotions as politically structured configurations produced by asymmetrical power relations rather than individual responses. This inhibitory effect is consistent with the role of repression in context‐specific activism: for participants under active political repression, fear and hopelessness reflect real threat appraisals rather than abstract states (Pearlman, 2013). The suppression pattern in the mediation analysis, a negative direct effect alongside a positive indirect effect, suggests fear inhibits action when experienced as paralyzing but facilitates it when reframed as a legitimate response to danger; across pathways, material damage emerged as a consistent mediator.

### The moral geography of violence: Material damage as the psychological hinge

Across models, material damage was the most consistent mediator of the association between emotions and activism, functioning as a central node in representations of political violence. Historical examples (e.g. Black Panthers, Zapatistas) received high consensus, while human and symbolic damage showed greater variability, reflecting cognitive polyphasia (de‐Graft Aikins, 2012; Moscovici, 2008). Material damage is cognitively framed as targeting structures rather than individuals, enabling support for disruptive action while maintaining moral boundaries around harm (Haidt, 2012; Rai & Fiske, 2011), a compromise between resistance and harm avoidance. Geopolitical patterns further illuminate this dynamic: participants from the Global South and Eastern Europe, contexts marked by histories of domination, showed higher acceptance of material damage, reflecting a ‘geography of contestation’ that challenges Eurocentric frameworks pathologizing resistance while normalizing state violence (Barkawi & Laffey, 2006; Becker & Tausch, 2017; Quijano, 2000).

### Symbolic damage and the expanding concept of violence

Symbolic damage emerged as a representational outlier, showing near‐zero correlations with other violence evaluation factors and uniquely predicted by self‐evaluative emotions such as guilt, shame, and pride, suggesting it occupies a distinct representational space centred on identity, memory and epistemic resistance rather than instrumental disruption. Actions like removing colonial statues or defacing monuments target representations while also producing material and epistemic ruptures, challenging dominant narratives that sustain power relations (Mignolo, 2007, 2009): guilt and shame signal recognition of complicity, while pride reflects alignment with resistance (Smith, 2019; Tuck & Yang, 2012). Its isolation also exposes limits in conventional definitions of violence, as it is experienced not as harm to persons or property but as disruption of meaning systems, resonating with Fanon's (1967) analysis of decolonization.

### Emotional profiles and the complexity of political subjectivity

Cluster analysis identified five distinct emotional profiles varying systematically in support for activism and acceptance of political violence across geopolitical regions and ideological orientations of the participants.

Crisis‐Driven Mobilizers (19.4%) reflect morally constrained mobilization: motivated by anger and hope in response to perceived crisis, but bounded by moral limits, reporting the lowest activism. Polyphasic Responders (23.2%) showed the highest acceptance of both material and human damage, suggesting that emotional plurality enables navigation of moral ambiguity; the coexistence of guilt and pride alongside anger and fear may reflect a form of ‘tragic activism’, acknowledging the ethical dilemmas inherent in resistance while remaining politically committed. Empowered Resisters (19.4%) showed among the highest levels of activism, suggesting that a configuration integrating anger and hope without action‐inhibiting self‐evaluative emotions or fear is associated with sustained engagement, though this does not imply that guilt is uniformly inhibitory, as guilt directed at ingroup obligations may function as a motivating force in other contexts (Shepherd et al., 2011; Wohl et al., 2006). This pattern is consistent with research showing that inclusive victimhood, acknowledging the suffering of both ingroup and outgroup, can facilitate rather than inhibit political mobilization (Cohrs et al., 2015). Disengaged Sceptics (9.7%) and Cautious Conformists (28.4%) both showed correspondingly low activism, representing either political apathy or a form of moral discomfort not associated with political engagement in this sample.

Within this sample, the observed regional associations are consistent with, though not confirmatory of, regional histories of political repression, state violence and collective action; future research with regionally stratified samples would be needed to establish whether these patterns generalize.

### Theoretical contributions

This study makes three core theoretical contributions, together with one more tentative, exploratory observation. First, we extend SRT by demonstrating that affective polyphasia operates alongside cognitive polyphasia as a functional resource for navigating moral complexity, Moscovici (2008), Jovchelovitch (2008), and de‐Graft Aikins (2012) conceptualized cognitive polyphasia as the coexistence of contradictory rationalities, a feature rather than a flaw of social thought, and our findings show this extends to the affective domain: individuals simultaneously experience anger and hope, guilt and pride, fear and determination, exemplified by the anger–hope dyad's distinct but complementary temporal orientations and motivational logics.

We also contribute to collective action research: our findings are consistent with the proposition that emotions may mobilize activism not through direct motivational force but through their anchoring in socially shared evaluations of protest legitimacy, refining SIMCA (Van Zomeren et al., 2008). In this cross‐sectional sample, transformative emotions were fully mediated by cognitive evaluations, self‐evaluative emotions were associated with activism through both direct identity processes and indirect cognitive appraisals, and threat emotions displayed a suppression pattern consistent with their ambivalence, challenging models that treat emotions as uniform motivational forces.

In addition, and more tentatively, given the low internal reliability of the Violence as a Tool factor (*α* = .45), which is retained for descriptive purposes only and excluded from all inferential analyses, the descriptive correlations suggest a differential relationship of power‐critical representations and moral values with this factor (*r* = .32 vs. *r* = .01), despite these dimensions being moderately correlated (*r* = .41), a functional rather than conceptual distinction. We advance the following as an interpretive hypothesis rather than a confirmed finding: moral values, as measured here, may express universal normative commitments that apply symmetrically across actors, constraining rather than licensing action, whereas power‐critical representations may instead encode a relational and asymmetric understanding of how dominant institutions define, frame and delegitimise resistance, such that violence by marginalized actors becomes structurally intelligible as a rational response to asymmetric power rather than a moral exception. Drawing on Hodges and Geyer's (2006) concept of value pragmatics, power‐critical representations may reorder the hierarchy of competing moral vocabularies, making structural justice a more salient evaluative criterion than procedural constraint: endorsement of Violence as a Tool may therefore reflect less the strength of one's ethical principles than how one represents the power relations within which violence occurs. Given the low reliability of this factor, we treat this pattern as a hypothesis to be confirmed with a more robust measure of instrumental attitudes towards violence.

Finally, we contribute to debates about the legitimacy of political violence by showing that evaluations of violence are structured by geopolitical context, colonial legacies and asymmetrical power relations: material damage functions as a psychological hinge enabling support for disruptive action while maintaining moral boundaries around human harm, while symbolic damage occupies a distinct representational space challenging the epistemic foundations of domination. These findings align with decolonial scholarship (Mignolo, 2007; Smith, 2019; Tuck & Yang, 2012) and critiques of Eurocentric frameworks that pathologize resistance while naturalizing state violence (Theodossopoulos, 2017); our contribution is to document how participants navigate these asymmetries, not to adjudicate between normative frameworks.

The present study is grounded in Social Representations Theory and informed by decolonial scholarship, shaping key design choices including the selection of diverse resistance cases, particularly from the Global South, anti‐colonial and Indigenous contexts, often underrepresented in political psychology. We have sought to distinguish our analytical framework from participants' own representations and to ground all empirical claims in the data.

## LIMITATIONS AND FUTURE DIRECTIONS

Our sample, while geographically diverse, was recruited through purposive convenience and snowball sampling and is not representative of broader populations. Participants were predominantly young (*M* = 29.15), educated and politically engaged, with a marked left‐leaning skew (45.0% vs. 14.4% right), likely inflating support for activism and the prevalence of mobilizing profiles over disengaged ones. Socioeconomic class was not systematically measured, so we cannot assess whether the emotional profiles reflect class‐specific or cross‐class patterns. The geographical distribution was also uneven, with underrepresentation of Sub‐Saharan Africa (*n* = 12) and Latin America (*n* = 8), limiting generalizability and precluding meaningful cross‐regional comparison for these groups.

The cross‐sectional design precludes causal inference: the mediation analyses' ordering reflects theoretical assumptions rather than temporal sequence, and it is equally plausible that activism shapes emotional repertoires as the reverse. The absence of measurement invariance testing across regions further limits cross‐regional comparisons, since differences in profile prevalence may reflect genuine variation or differential item functioning. The English‐only survey may have introduced systematic response biases, potentially underrepresenting non‐Western political imaginaries and resistance traditions.

Future research should address these limitations through longitudinal designs examining whether emotional repertoires precede or follow shifts in activism, regionally stratified sampling with translated instruments enabling measurement invariance testing, incorporation of socioeconomic class, experimental or vignette‐based designs manipulating the framing of resistance contexts (e.g. state repression), and qualitative follow‐up enriching understanding of the more complex profiles (Polyphasic Responders, Cautious Conformists).

## CONCLUSION

This study invites a reconceptualization of violence as encompassing not only physical force but also epistemic rupture: the disruption of taken‐for‐granted meanings, the challenging of authoritative narratives, and the assertion of alternative historical truths. Violence, in this expanded sense, is always relational and representational, embedded within asymmetrical power structures and negotiated through socially shared systems of meaning. Understanding political violence therefore requires attending not only to acts themselves but to the emotional, cognitive and moral frameworks through which those acts are interpreted, evaluated, and (de)legitimized. It is through these polyphasic systems, integrating anger and hope, material and symbolic resistance, moral constraint and political necessity, that individuals navigate the moral complexity of political violence, revealing the representational and affective architecture through which resistance is evaluated, justified and sustained.

## AUTHOR CONTRIBUTIONS

**Subas Amjad Ali:** Conceptualization; investigation; writing – original draft; methodology; visualization; writing – review and editing; data curation; formal analysis; validation; supervision. **Anderson da Silveira:** Methodology; visualization; formal analysis. **Kata Bődi:** Visualization; validation; data curation. **Hanan Omar Abdallah Zaid Alkilani:** Validation; data curation. **Sara Bigazzi:** Conceptualization; investigation; validation; methodology; supervision; writing – original draft; formal analysis; writing – review and editing.

## CONFLICTS OF INTEREST STATEMENT

The authors declare no conflicts of interest.

## Supporting information


Supplementary Note 1.

## Data Availability

The data that support the findings of this study are available from the corresponding author upon reasonable request.
