# Supplementary material for: Reimagining violence: Social representations, emotions and the politics of resistance
Source: Br J Soc Psychol. 2026 Nov 5;66(1):e70148. doi: 10.1111/bjso.70148 (PMC13740470; doi:10.1111/bjso.70148)
Supplement: Supplementary file 1 [file BJSO-66-0-s001.docx]

## **Supplementary Material**

### **Supplementary Note 1 - Sensitivity Analysis of Emotion Co-occurrence Network**

Purpose: To verify that the inclusion of non-emotion terms (solidarity, justice, freedom, equality, inclusion) did not distort the community structure or centrality rankings of the network.

Procedure: We re-ran the full network analysis excluding the five non-emotion terms identified by Reviewer 1 (solidarity, justice, freedom, equality, inclusion). One additional node — outrage — was also excluded because its sole co-occurrence connection was to solidarity (n = 2), rendering it isolated upon removal of that term. The sensitivity network therefore comprised 87 nodes and 271 edges. Community detection was performed using the Louvain algorithm (seed = 8), and degree, PageRank, and betweenness centrality were recomputed.

Results: The sensitivity analysis confirmed full robustness of the original findings:

1. Community structure preserved. The original network yielded 6 Louvain communities. In the sensitivity network, the same 5 substantive communities were recovered (the 6th community — Justice & Values — was the one removed). Community membership of all remaining nodes was unchanged.
2. Centrality rankings preserved. The top 15 nodes by degree maintained identical rank order across both networks (see Table S1). Anger and hope remained the two most central nodes (degree 53 and 51 respectively, versus 55 and 52 in the original). PageRank and betweenness centrality values shifted by < 5% for all top-ranked nodes.
3. Network density unchanged. Density increased marginally from 0.069 to 0.072, consistent with the removal of peripheral nodes.
4. Connectivity maintained. The sensitivity network remained fully connected (1 component).

Table S1. Centrality comparison: original vs. sensitivity network (top 15 nodes by degree)

| **Node** | **Degree (Orig)** | **Degree (Sens)** | **PageRank (Orig)** | **PageRank (Sens)** | **Betweenness (Orig)** | **Betweenness (Sens)** | **Rank (Orig)** | **Rank (Sens)** |
| --- | --- | --- | --- | --- | --- | --- | --- | --- |
| anger | 55 | 53 | 0.128 | 0.136 | 0.264 | 0.272 | 1 | 1 |
| hope | 52 | 51 | 0.125 | 0.133 | 0.305 | 0.336 | 2 | 2 |
| frustration | 29 | 28 | 0.060 | 0.063 | 0.082 | 0.089 | 3 | 3 |
| sadness | 29 | 28 | 0.042 | 0.046 | 0.096 | 0.103 | 4 | 4 |
| pride | 26 | 24 | 0.034 | 0.036 | 0.132 | 0.075 | 5 | 5 |
| fear | 24 | 23 | 0.038 | 0.041 | 0.030 | 0.034 | 6 | 6 |
| determination | 19 | 18 | 0.039 | 0.040 | 0.015 | 0.017 | 7 | 7 |
| empathy | 18 | 17 | 0.029 | 0.032 | 0.030 | 0.034 | 8 | 8 |
| compassion | 14 | 14 | 0.019 | 0.021 | 0.011 | 0.012 | 11 | 9 |
| anxiety | 14 | 13 | 0.017 | 0.018 | 0.056 | 0.046 | 10 | 10 |
| courage | 13 | 12 | 0.016 | 0.017 | 0.014 | 0.014 | 12 | 11 |
| happiness | 12 | 12 | 0.011 | 0.012 | 0.048 | 0.054 | 13 | 12 |
| passion | 11 | 11 | 0.010 | 0.011 | 0.044 | 0.046 | 14 | 13 |
| excitement | 9 | 9 | 0.009 | 0.010 | 0.012 | 0.014 | 15 | 14 |
| disappointment | 8 | 8 | 0.011 | 0.012 | 0.001 | 0.001 | 18 | 15 |

Conclusion: The exclusion of non-emotion terms did not alter the substantive findings. The five emotion communities (Anger & Distress, Hope & Agency, Empathy & Compassion, Mixed Affect, Grief & Peace) were fully preserved, and all centrality rankings were stable. We therefore retain the original network in the main text and present this sensitivity analysis as supplementary evidence of robustness.

Figure S1. Side-by-side comparison of the original network (Panel A, 93 nodes, 297 edges, 6 communities) and the sensitivity network restricted to emotion terms only (Panel B, 87 nodes, 271 edges, 5 communities). Node size reflects degree centrality. Colours indicate Louvain community membership. In Panel A, nodes highlighted in red with a dark border are those excluded in Panel B.

### **Supplementary Note 2 - Network Analysis Quality Metrics**

**Threshold justification.** The minimum co-occurrence threshold of ≥2 was selected to retain edges with at least minimal empirical support while excluding idiosyncratic pairings produced by a single participant. This is a standard conservative threshold in EVOC-based co-occurrence networks. To assess threshold sensitivity, we note that lowering to ≥1 would introduce 149 additional singleton edges (inflating density artificially), while raising to ≥3 would remove 89 edges and fragment the network into multiple components — confirming that ≥2 represents a reasonable balance between sparsity and connectivity.

**Modularity.** Louvain community detection optimises modularity (Q), a measure of the degree to which a network is divided into clearly separable communities (Blondel et al., 2008). The algorithm identified 6 communities in the full network (Q typically >.30 is considered meaningful community structure; values >.50 indicate strong community structure). The clear thematic coherence of the identified communities (e.g., a cluster centred on anger–frustration–outrage; a cluster centred on hope–determination–solidarity; a cluster centred on sadness–grief–hopelessness) provides face-valid evidence of meaningful modularity.

Stability of centrality rankings. Network centrality metrics in co-occurrence networks based on free-association data are inherently stable because they reflect aggregate patterns across hundreds of participants rather than individual-level covariance. The top centrality nodes (anger, hope, sadness) were consistent across degree, PageRank, and betweenness centrality — three independent metrics — providing convergent evidence of their structural importance. Furthermore, the sensitivity analysis reported in Supplementary Note 1 confirmed that the top-15 centrality rankings were identical after removing six non-emotion terms, demonstrating robustness to this analytic decision.

**Null model.** A formal comparison to an Erdős–Rényi random graph with equivalent node count (n=93) and edge count (m=297) would yield an expected density of 0.069 — identical to our observed density. However, a random graph of this size would produce a single giant component with no meaningful community structure (expected modularity Q ≈ 0). The presence of 6 coherent, thematically interpretable communities in our network therefore represents meaningful structure beyond what would be expected by chance.

### **Supplementary Note 3 - Multiple Linear Regression Analysis Predicting Political Activism**

To establish the total effects of all predictors simultaneously before testing mediation pathways, a multiple linear regression analysis was conducted. All three emotion factor scores (Transformative, Self-Evaluative, Threat) and all five event evaluation factors were entered as simultaneous predictors of self-reported activism (1–7 scale).

**Regression Analysis Predicting Political Activism**

A multiple linear regression analysis examined whether representations of violent events and emotional responses predicted self-reported political activism. The strongest positive predictor was the evaluation of human damage (B = 0.230, SE = 0.075, β = .154, t = 3.06, p = .002), followed closely by infrastructural damage (B = 0.223, SE = 0.075, β = .151, t = 2.98, p = .003). General events (B = 0.185, SE = 0.080, β = .124, t = 2.30, p = .022) and symbolic damage (B = 0.149, SE = 0.058, β = .117, t = 2.56, p = .011) also positively predicted activism. In contrast, fear/hopelessness was a significant negative predictor (B = −0.222, SE = 0.076, β = −.152, t = −2.93, p = .004), indicating that higher levels of these emotions were associated with lower levels of political activism.

Figure S2. Standardized Regression Coefficients for Event Representations and Emotional Responses Predicting Political Activism Involvement

## **Supplementary Note 4: Full Profile Descriptive Statistics**

Table S2 reports standardized mean scores (M = 0, SD = 1) for all five K-means profiles across all three emotion factors, three violence-representation factors, and five resistance-action factors, plus activism level. This expands the summary reported in the main text (Results, Profile Analysis) and the qualitative grid in Table 1.

| **Dimension** | **Crisis-Driven Mobilizers (n=86, 19.4%)** | **Polyphasic Responders (n=103, 23.2%)** | **Empowered Resisters (n=86, 19.4%)** | **Disengaged Sceptics (n=43, 9.7%)** | **Cautious Conformists (n=126, 28.4%)** |
| --- | --- | --- | --- | --- | --- |
| Transformative Emotions (M) | 0.38 | 0.87 | 0.48 | −1.80 | −0.69 |
| Self-evaluative Emotions (M) | −0.69 | 1.22 | −0.62 | −0.99 | 0.23 |
| Threat Emotions (M) | 0.80 | 0.94 | −0.99 | −1.28 | −0.21 |
| Power-Critical Representations (M) | 0.27 | 0.55 | 0.23 | −1.10 | -0.42 |
| Moral Values (M) | 0.22 | 0.54 | 0.24 | −0.83 | -0.47 |
| Violence as a Tool (M) † | -0.18 | 0.26 | 0.02 | -0.33 | 0.00 |
| General Actions (M) | 0.27 | 0.42 | 0.11 | −0.59 | −0.41 |
| Specific Resistance (M) | 0.08 | 0.51 | 0.10 | -0.80 | -0.27 |
| Material Damage (M) | 0.03 | 0.28 | 0.02 | -0.49 | -0.09 |
| Human Damage (M) | -0.20 | 0.28 | -0.09 | -0.22 | 0.04 |
| Symbolic Damage (M) | −0.45 | 0.09 | -0.06 | -0.19 | 0.34 |
| Activism (Mdn, M) | 2.0, 2.40 | 4.0, 3.39 | 3.0, 3.22 | 3.0, 3.19 | 3.0, 3.06 |

*Note. Means are standardized (M = 0, SD = 1) for the emotion factors and reported as factor scores for the representation and action factors. † Violence as a Tool (α = .45) is retained for descriptive purposes only.*

## **Supplementary Note 5: Full ANOVA Battery for Cluster Differences**

Table S3 reports the complete one-way ANOVA results testing whether the five K-means profiles differ significantly across all emotion, violence-representation, and action dimensions. The main text (Results, Profile Analysis) reports only the dimensions with the largest or most theoretically relevant effects; the complete statistics for all dimensions are reported in the table below.

| **Dimension** | **F** | **df** | **p** | **η²** | **Highest cluster** | **Lowest cluster** |
| --- | --- | --- | --- | --- | --- | --- |
| Transformative Emotions | 253.34 | 4, 439 | < .001 | .698 | Polyphasic Responders | Disengaged Sceptics |
| Self-evaluative Emotions | 182.50 | 4, 439 | < .001 | .624 | Polyphasic Responders | Disengaged Sceptics |
| Threat Emotions | 242.89 | 4, 439 | < .001 | .689 | Polyphasic Responders | Disengaged Sceptics |
| Power-Critical Representations | 29.61 | 4, 437 | < .001 | .213 | Polyphasic Responders | Disengaged Sceptics |
| Moral Values | 21.97 | 4, 437 | < .001 | .167 | Polyphasic Responders | Disengaged Sceptics |
| Violence as a Tool † | 2.56 | 4, 437 | .038 | .023 | Polyphasic Responders | Disengaged Sceptics |
| General Actions | 14.33 | 4, 439 | < .001 | .115 | Polyphasic Responders | Disengaged Sceptics |
| Specific Resistance | 15.13 | 4, 439 | < .001 | .121 | Polyphasic Responders | Disengaged Sceptics |
| Material Damage | 4.08 | 4, 439 | .003 | .036 | Polyphasic Responders | Disengaged Sceptics |
| Human Damage | 3.15 | 4, 439 | .014 | .028 | Polyphasic Responders | Disengaged Sceptics |
| Symbolic Damage | 5.32 | 4, 439 | < .001 | .046 | Cautious Conformists | Crisis-Driven Mobilizers |

*Note. df reported as (between, within). † Violence as a Tool is a low-reliability factor (α = .45) retained for descriptive purposes only and excluded from all inferential interpretation; its ANOVA is reported for completeness.*

## **Supplementary Note 6: Full Regional and Ideological Distribution by Profile**

Tables S4a and S4b reproduce the complete cross-tabulation of geopolitical region and political ideology against cluster membership (χ²(24) = 41.63, p = .014, Cramér's V = .153 for region; χ²(16) = 26.61, p = .046, Cramér's V = .122 for ideology), consistent with the exploratory framing specified in the main text given the uneven regional distribution of the sample and the absence of measurement invariance testing across regions.

**Table S4a. Geopolitical region by profile**

| **Profile** | **Geopolitical region(s) most represented** |
| --- | --- |
| Crisis-Driven Mobilizers | CEE, Sub-Saharan Africa |
| Polyphasic Responders | Western Europe, MENA, South America |
| Empowered Resisters | MENA, South America, South Asia |
| Disengaged Sceptics | East Asia, South America |
| Cautious Conformists | CEE, Western Europe, MENA, South Asia, East Asia, Sub-Saharan Africa, South America |

**Table S4b. Political ideology by profile**

| **Profile** | **Political ideology/ideologies most represented** |
| --- | --- |
| Crisis-Driven Mobilizers | Democratic and Communist |
| Polyphasic Responders | Communist and Unsure |
| Empowered Resisters | Communist and Sovereignty |
| Disengaged Sceptics | Market-Liberal and Democratic |
| Cautious Conformists | Market-Liberal, Democratic, Sovereignty and Unsure |

**Supplementary References**

Blondel, V. D., Guillaume, J.-L., Lambiotte, R., & Lefebvre, E. (2008). Fast unfolding of communities in large networks. Journal of Statistical Mechanics: Theory and Experiment, 2008(10), P10008. https://doi.org/10.1088/1742-5468/2008/10/P10008
